# Effect of yoga on academic performance in relation to stress

**DOI:** 10.4103/0973-6131.53860

**Published:** 2009

**Authors:** Amit Kauts, Neelam Sharma

**Affiliations:** Principal, MGN College of Education, Jalandhar, India; 1 2B, Old Baradari, Jalandhar City, Punjab, India

**Keywords:** Academic, performance, stress, yoga, yoga module

## Abstract

**Background:**

Academic performance is concerned with the quantity and quality of learning attained in a subject or group of subjects after a long period of instruction. Excessive stress hampers students’ performance. Improvement in academic performance and alertness has been reported in several yogic studies.

**Aims and Objectives:**

The main objective of the study was to assess the effect of yoga on academic performance in relation to stress.

**Materials and Methods:**

The study started with 800 adolescent students; 159 high-stress students and 142 low-stress students were selected on the basis of scores obtained through Stress Battery. Experimental group and control group were given pre test in three subjects, i.e., Mathematics, Science, and Social Studies. A yoga module consisting of yoga asanas, pranayama, meditation, and a value orientation program was administered on experimental group for 7 weeks. The experimental and control groups were post-tested for their performance on the three subjects mentioned above.

**Results:**

The results show that the students, who practiced yoga performed better in academics. The study further shows that low-stress students performed better than high-stress students, meaning thereby that stress affects the students’ performance.

## INTRODUCTION

Academic achievement is an attained ability or degree of competence in school task, usually measured by standardized tests and expressed in grades or units based on norms derived from a vide sampling of pupils’ performance.[1] Studies reveal that even low or moderate levels of stress can interfere with task performance.[2,3] Cognitive reactions of stress result in the inability to concentrate.[4]

Yoga, which is a way of life, is characterized by balance, health, harmony, and bliss.[5] Meditation, being part of yoga, which is the seventh limb of Ashtanga Yoga[6] -a state of alert rest as stated by Maharishi Mahesh Yogi,[7] who founded a new technique of meditation, popularly known as transcendental meditation. By practicing yoga, a person is supposed to reach a state of mental equanimity, where responses to favorable or unfavorable external events are well under the individual’s control, and responses are moderate in intensity.[8] The science of yoga is a powerful stream of knowledge, which enables the practitioners to achieve radiant physical health, serene mind, continues spiritual uplift, and creates the ability for harmonious social living.[9]

Hatha yoga practices, like asanas (i.e., postures), pranayama (i.e., breathing practice intended to influence vital forces), kriyas (cleaning processes), mudras (i.e., certain interval attitudes), and bandhans (i.e., neuromuscular locks) are mostly taught as physical practices. While various meditational techniques work at the mental level, all these practices are intended to develop a certain type of awareness within oneself, which in turn brings about a change in emotional and visceral functions, and through them, a change in intellectual and somatic functions of the individual takes place.[10]

Six months of yogic practices (meditation, asanas, and pranayama) brings a feeling of well-being, a reduction in body weight, increased vital capacity, acceleration in endocrinal functions, and improvement in memory.[11] Three months practice of Savasana has demonstrated an improvement in 86 patients, who had problem of headache, insomnia, and nervousness.[12] Udupa *et al*.[13] revealed that yoga has the potential to influence the stress disorder and it helps the sufferer to achieve physical and metabolic stability. Sahasi *et al*.[14] has demonstrated the effectiveness of yogic techniques in the management of anxiety and reported increased attention/concentration.

Yoga through its techniques of meditation, asanas, and pranayama yields a positive effect in the management of stress in adolescents.[15] The processing of sensory information at the thalamic level is facilitated during the practice of pranayama[16] and meditation.[17,18] These two practices along with physical postures (asanas), cleansing practices, devotional sessions, and lectures on the theory and philosophy of yoga were focused to bring about an improvement in the steadiness of school students following 10 days of practice. This improvement was believed to be due to improved eye-hand coordination, attention, concentration, and relaxation.[19]

In one study, it is found that a 4-week program of yogasanas and meditation lowers the aggressive behavior of students.[20] Another study has reported that meditation (a) reduced problems related to maladaptive behaviors, (b) increased emotional and physical health and psychological well-being, (c) reduced the frequency of thought, (d) reduced substance abuse, and (e) generally improved the quality of life.[21]

Transcendental meditation reduces stress[22] and improves academic performance.[23–32] Chanting “Om” mentally causes increased alertness,[33] and the practice of yoga brings improvement in competitive performance.[34]

The research done by Mind/Body Institute, Harvard Medical[35] School, and Bruce D’ Hara and his team at the University of Kentucky in Lexington, U.S., revealed a positive influence of meditation on brain functioning and performance.[36]

The present study examines whether there is an effect of yoga on the academic performance of adolescent students in relation to their stress. With this background, the present study was conducted to find the following: (1) Is there any effect of yoga on the academic performance in Mathematics, Science, and Social Studies in relation to stress? (2) Is there any effect of yoga on the academic performance in the three subjects combined in relation to stress.

## MATERIALS AND METHODS

### Subjects

The study was conducted in eight public schools of Jalandhar, Punjab. Bisht Battery of Stress Scale (BBSS)[37] was administered on 800 students of Class 9. The participants were 400 boys and 400 girls, with ages ranging from 14 to 15 years. BBSS was administered to identify two stress levels of the students, i.e., high stress and low stress. This test was developed for the measurement of 13 types of stress. Out of 13 scales, two scales, i.e., scale of academic stress and scale of achievement stress were selected. These scales were consisted of 52 and 80 items, respectively, which were 132 in total. Each item is of statement type (closed), to which students were to answer by ticking their option prescribed on the answer sheet. The students were assembled in a hall and made to sit in rows. Booklets containing statement items along with answer sheets were distributed to each student. Instructions were delivered by the investigator. Statements were written in Hindi. Meaning of difficult words was also explained. The students were told to finish their test within an hour.

The scoring was done as prescribed in the manual. On the basis of their stress scores arranged in ascending order, top 30% (i.e., 240) subjects were identified as students with low stress and bottom 30% (i.e. 240) students were identified as student with high stress. Out of these students, 50% of them were kept in experimental group and another 50% in control group. Finally 30% subject [high stress (exp) = 89 + low stress (exp) = 75 + high stress (control): 70 + low stress (control) = 67] were selected. Pretest was conducted in three subjects, i.e. Mathematics, Science, and Social Studies for both the groups. Ultimately, 301 subjects (116 girls and 185 boys) were selected for the present study.

### Ethics

A code was provided to the students at the time of pretest to keep their personal identity closed. Their achievement scores were exclusively used for the research purpose and were not disclosed to their educational institutions. The project was approved by the Institutional Ethics Committee, and the signed informed consent was obtained from the school principal.

### Assessments

Bisht Battery of Stress Scale was used to identify different levels of stress among the students, i.e., high stress and low stress. This was done before the start of experiment. Details of its administration ARE mentioned above under the heading “Subjects.”

Yoga module was used as an intervention treatment for the experimental group for an hour daily in the morning for 7 weeks.

Academic performance test was used as a pretest and posttest for the experimental as well as control groups to assess the effect of yoga module on the academic performance of the experimental group and to compare it with the control group, who never practiced yoga module.

### Intervention

A yoga module [yogasana + pranayama + meditation + prayer + value orientation program] was shared daily for an hour in the morning with the experimental group for 7 weeks. Same academic performance test was administered on the both groups as a posttest.

### Statistical analysis

To study an effect of yoga and stress on the academic performance, 2 × 2 factorial design (ANOVA) was employed on the gain scores of academic performance, wherein stress is a classificatory variable and is studied at two levels, i.e., students with high stress and students with low stress. Yoga module, taken as treatment variable, was given to the experimental group. Data are displayed in Tables 1–4.

## RESULTS

Table 2 reveals that F-ratio for the difference between means of experimental group (yoga) and control group on the gain scores of combined academic performance (in Mathematics, Science, and Social Studies) is found to be significant at the 0.01 level of confidence, which indicates that students of the experimental group and the control group differ on the gain scores of academic performance. Table 1 depicts means and SDs of combined academic performance gain scores of three subjects, in which the mean of experimental group [M_exp_= 32.63] is found to be greater than that of the control group [M_c_= 22.44], meaning thereby that those students who experienced yoga module performed better than those who never experienced it.

Table 2 further shows that F-ratio for the difference between means of high stress group and low stress group on the gain scores of combined academic performance was found to be significant at the 0.05 level of confidence, indicating that academic performance differs among students with high stress and students with low stress. Table 1 shows that the group means of students with low stress [M_LS_= 30.24] is greater than the group mean of the students with high stress [M_HS_= 24.83], meaning thereby that students with low stress performed better than the students with high stress. Thus, this study reveals that the high stress affects students’ performance negatively, and this result is in tune with the inverted U-shaped model of stress of learning.[38] Table 2 further depicts no interaction between yoga intervention treatment and stress on the gain scores of academic performance in three subjects combined. After seeing the positive effect of yoga on the three subjects combined, we thought to have a deeper analysis to study the effect of yoga on different subjects separately. In this context, the data are presented in Tables 3 and 4. shows that F-ratio for the difference between means of high stress group and low stress group on the gain scores of academic performance in Mathematics, Science, and Social Studies (separately) is found to be significant at 0.01 level of confidence, which indicates that students of the experimental group and the control group differ on the gain scores of academic performance in the three subjects. Table 3 gives the details of means and SDs, which depicts that the experimental group showed higher group mean [M_Soc. St._=5.11, M _Maths_=5.53, M_Sc_=5.28] than control group [M_Soc. St._=2.82, M _Maths_=2.83, M_Sc_=3.37]. It is inferred that the students who experienced yoga module performed better than those students who never experienced it.

Further, Table 4 shows that F-ratio for the difference between means of high stress group and low stress group on the gain scores of academic performance in all three subjects (separately) is not found to be significant even at the 0.05 level of confidence.Table 3 gives details which reveal that means of academic performance in all three subjects in two groups, i.e., high stress and low stress, are comparable.

**Table 1:** Means and SDs of academic performance gain scores of three subjects combined

	EXP (Yoga intervention)	C (without yoga intervention)	
HS	–	–	M_HS_ = 24.83
	X_1_ = 30.99	X_2_ = 18.68	
	σ_1_ = 17.50	σ_2_ = 13.14	
LS	–	–	M_LS_ = 30.24
	X_3_ = 34.28	X_4_ = 26.20	
	σ_3_ = 21.56	σ_4_ = 18.72	
	M_EXP_ = 32.63	M_C_ = 22.44	

**Table 2:** Summary of ANOVA on the academic performance gain scores of three subjects combined in relation to stress and yoga intervention

Source of variance	df	SS	MSS	F-ratio
Yoga “A“	1	7669.77	7669.77	23.39**
Stress “B”	1	1802.3	1802.3	5.50*
Interaction	1	575.68	575.68	1.75
Within	296	97702.53	330.07	-
Total		107750.28		-

* Significant at the 0.05 level of confidence;

** Significant at the 0.01 level of confidence

**Table 3:** Means and sd‘s of academic performance gain scores of social studies, mathematics and science in high stress and low stress group with and without yoga intervention

	EXP			Control			
	Social St.	Maths	Science	Social st.	Maths	Science	
HS	–	–	–	–	–	–	M_HS_ S. St = 4.26
	X_1_ = 5.74	X_1_ = 5.73	X_1_ = 5.52	X_2_ = 2.78	X_2_ = 2.57	X_2_ = 3.28	M_HS_ Maths = 4.15
	σ_1_ = 6.85	σ_1_ = 3.93	σ_1_ = 3.84	σ_2_ = 3.43	σ_2_ = 3.43	σ_1_ = 3.75	M_HS_ Sci = 4.4
LS	–	–	–	–	–	–	M_HS_ S. St = 3.67
	X_3_ = 4.48	X_3_ = 5.34	X_3_ = 5.04	X_4_ = 2.86	X_4_ = 3.09	X_4_ = 3.46	M_LS_ Maths = 4.21
	σ_3_ = 4.07	σ_3_ = 4.24	σ_4_ = 4.004	σ_4_ = 3.59	σ_4_ = 3.70	σ_4_ = 3.74	M_LS_ Sci = 4.25
	M_EXP_ Social St. = 5.11	M_EXP_ Maths= 5.53	M_EXP_ Sci. = 5.28	Mc S. St. = 2.82	Mc Maths = 2.83	Mc Sci. = 3.37	

**Table 4:** Summary of anova on the academic performance scores of social studies, mathematics and science in relation to stress and yoga intervention

Source of variance	df	S.S.			M.S.S.			F-ratio			
		Social studies	Maths	Science	Social studies	Maths	Science	Social studies	Maths	Science
Yoga ‘A’	1	408.65	556.34	277.04	408.65	556.34	277.04	17.13*	37.02*	18.58*
Stress ‘B’	1	39.08	0.247	4.28	39.08	0.247	4.28	1.63	0.016	0.28
Interaction	1	25.95	14.92	6.04	25.95	14.92	6.04	1.08	0.99	0.40
Within	296	7108.37	4479.14	4441.06	24.01	15.13	15.00	–	–	–
Total		7582.05	5050.65	4728.42	–	–	–	–	–	–

* Significant at the 0.01 level of confidence.

## DISCUSSION

**Figure 1:**
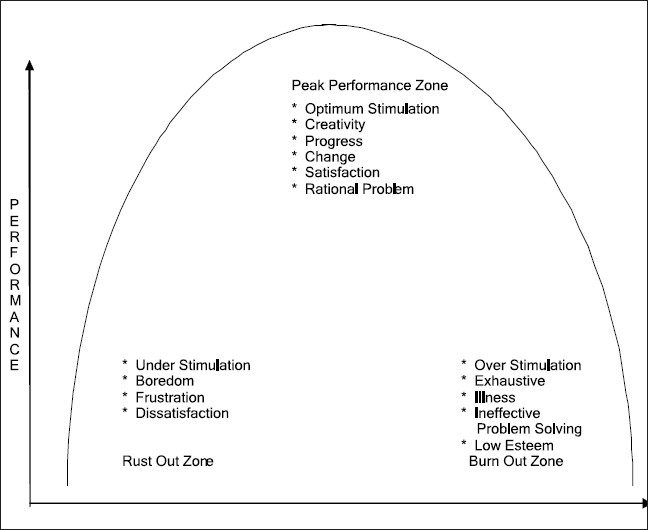
Figure showing effect of stress on performance *(Source: - Kindler and Ginsberg (1990))*

The findings of this study reveal that the students who experienced yoga module performed better in overall academics as well as in their separate subjects than those students who did not experience yoga module. The results are in tune with the earlier studies, which found that meditation, practiced over long periods, produces definite changes in perception, attention, and cognition.[37] Other study showed that yoga techniques are helpful in management of anxiety and improvement in concentration.[14] Other researchers found that Transcendental Meditation improves academic performance and enhances problem-solving ability.[23–32] Table 2 (a) shows that the students with high stress performed better in the subjects of Social Studies and Science. This result is in tune with the inverted U-shape model of stress and learning,[38] which explains that at first, performance improves as stress increases presumable because the stress is arousing or energizing. Beyond some point, though stress becomes distracting and performance actually drops as depicted in Figure 1.[39,40]

Further the findings reveal that excessive stress affects overall academic performance negatively, and this result is in tune with the earlier studies, which conclude that excessive stress is harmful to academic performance[41,42] and may lead to dropping out.[43,44] Research has demonstrated that high levels of stress can lead to hypervigilance (inability to focus attention) as arriving at a solution too quickly (premature closure).[45] Higher levels of stress reduced grade point average (GPA) among 146 college men and led to increased psychological and somatic symptomology.[46] When stress is perceived negatively or becomes excessive, students experience physical and psychological impairment.[47] Stress overloads our mental and physical resources and interferes with the effective use of our skills, and thus, affects negatively on the performance.[48]

Moreover, when academic performance in individual subjects was analyzed, the performance was comparable in high stress and low stress groups, but having values very close to significant values.

It may be concluded from the finding of the study that with the intervention of yoga, academic performance improves by optimizing the stress levels. So it is suggested that yoga module should become a regular feature in the schools.
